# Aspects cliniques et paracliniques de l´épilepsie de l´enfant à l´Hôpital de la Paix de Ziguinchor: étude documentaire

**DOI:** 10.11604/pamj.2020.37.387.21902

**Published:** 2020-12-30

**Authors:** Lamine Thiam, Ndiogou Seck, François Niokhor Diouf, Djibril Boiro, Babacar Niang, Salimata Diang Sagna, Adama Coundoul, Pape Mactar Faye, Moustapha Ndiaye, Amadou Lamine Fall, Ousmane Ndiaye

**Affiliations:** 1 Université Assane Seck de Ziguinchor, Hôpital de la Paix de Ziguinchor, Ziguinchor, Sénégal,; 2 Université Gaston Berger de Saint Louis, Hôpital régional de Saint Louis, Saint Louis, Sénégal,; 3 Hôpital Régional de Ziguinchor, Ziguinchor, Sénégal,; 4 Université Cheikh Anta Diop de Dakar, Hôpital Abass Ndao de Dakar, Dakar, Sénégal,; 5 Université Cheikh Anta Diop de Dakar, Hôpital d´Enfants Albert Royer de Dakar, Dakar, Sénégal,; 6 Hôpital Psychiatrique de Ziguinchor, Ziguinchor, Sénégal

**Keywords:** Épidémiologie, épilepsie, enfance, pédiatrie, Epidemiology, epilepsy, childhood, pediatrics

## Abstract

L´épilepsie pose un problème de santé publique au Sénégal. L´objectif de l´étude était de décrire les aspects cliniques et paracliniques de l´épilepsie de l´enfant à l´Hôpital de la Paix de Ziguinchor (HPZ). Il s´agissait d´une étude documentaire de dossiers d´enfants épileptiques, du 1^er^ janvier 2015 au 31 décembre 2018. Ont été inclus, les patients âgés ≤ de 15 ans, suivis pour épilepsie à l´HPZ. Etaient exclus, les dossiers incomplets. Cinquante-cinq (37 garçons et 18 filles) enfants ont été colligés; 70,9% avaient un âge < à 5 ans. L´âge moyen était de 4,3 ans. Les patients étaient originaires du milieu rural (60%) et de famille défavorisée (67,3%). Les crises d´épilepsie étaient généralisées (72,7%) et focales (27,3%). Dix-huit patients présentaient une épilepsie idiopathique, 17 une épilepsie non idiopathique. Les facteurs étiologiques étaient dominés par les anomalies de la grossesse et de l´accouchement (29,1%). L´épilepsie est fréquente à l´HPZ. Elle prédomine en milieu rural chez les garçons de moins de 5 ans, issus de famille défavorisée. Les crises généralisées tonico-cloniques et les crises focales sont les tableaux cliniques les plus fréquentes et les anomalies de la grossesse et de l´accouchement sont les étiologies les plus retrouvées.

## Abstract (en)

Epilepsy poses a public health problem in Senegal. The purpose of the study was to describe the clinical and paramedical features of epilepsy in children at the Ziguinchor Peace Hospital (ZPH). We conducted a literature review of the medical records of children with epilepsy, from January 1, 2015 to December 31, 2018. Patients aged < 15 years followed up for epilepsy at the ZPH were included. Incomplete medical records were excluded. Data from fifty-five (37 boys and 18 girls) children were collected; 70.9% of them were ≤5 years of age. Mean age of patients was 4.3 years. Patients were from rural (60%) and disadvantaged families (67.3%). Seizures were generalized (72.7%) and focal (27.3%). Eighteen patients had idiopathic epilepsy, 17 had non-idiopathic epilepsy. Etiological factors were dominated by abnormalities associated with pregnancy and childbirth (29.1%). Epilepsy is common at the ZPH. It is most common in rural areas among boys under the age of 5 years from disadvantaged families. Generalized tonic-clonic and focal seizures are the most frequent clinical state and abnormalities associated with pregnancy and childbirth are the most commonly found etiologies.

## Introduction

L´épilepsie est une maladie cérébrale caractérisée par une activité électrique anormale provoquant des convulsions ou un comportement inhabituel, des sensations et, parfois, une perte de conscience [1]. Elle a des conséquences neurologiques, cognitives, psychologiques et sociales et représente une part importante de la charge de morbidité mondiale, touchant près de 50 millions de personnes dans le monde [1]. Le nombre de personnes atteintes d´épilepsie devrait encore augmenter compte tenu de la hausse de la proportion croissante de personnes survivant à des traumatismes qui conduisent souvent à l´épilepsie, comme les traumatismes obstétricaux, les traumatismes cérébraux, les infections cérébrales et les accidents vasculaires cérébraux. Les conséquences physiques, psychologiques et sociales de l´épilepsie représentent un lourd fardeau pour les personnes qui en sont atteintes et leur famille. Partout dans le monde, les personnes épileptiques et leur famille sont victimes de stigmatisation et de discrimination, et vivent souvent des situations très difficiles en matière d´éducation, d´emploi, de mariage ou de santé reproductive. Près de 80% des personnes atteintes d´épilepsie vivent dans les pays en voie de développement [1]. Or, dans la plupart de ces pays à revenu faible, plus de 75% des personnes qui devraient bénéficier d´un traitement n´y ont pas accès ; ce chiffre est de 50% dans la plupart des pays en voie développement. Pourtant, les médicaments antiépileptiques sont efficaces et bon marché [1]. D'une manière générale et sans considération d'âge, la prévalence de l'épilepsie en Afrique subsaharienne varie entre 7 et 14,8‰ [2,3]. Elle concerne principalement les enfants. Au Sénégal, la prévalence de l´épilepsie était estimée à 14,2‰° dans la banlieue dakaroise en 2001. Dans cette étude, les auteurs avaient retrouvé 23,4% de toutes les personnes atteintes d'épilepsie qui n'avaient jamais reçu de traitement approprié [4]. Dans la région de Ziguinchor, aucune étude sur l´épilepsie, toutes tranches d´âge confondues n´a été réalisée à ce jour. La stigmatisation de la maladie, le manque de personnel qualifié, particulièrement de neurologues pédiatres, la quasi absence des appareils d´électroencéphalogramme (EEG) dans la région expliquent ce manque de données. L´avènement de l´Unité de Formation et de Recherche en sciences de la santé dans la région de Ziguinchor a permis le déploiement de plusieurs spécialistes dans ladite région dont un neuro-pédiatre. Il a été initié dans ce cadre une consultation hebdomadaire de neurologie pédiatrique au sein de l´Hôpital de la Paix de Ziguinchor (HPZ). L´objectif de l´étude était de décrire les aspects cliniques et paracliniques de l´épilepsie de l´enfant dans la cohorte de l´HPZ.

## Méthodes

**Nature, période, cadre de l´étude**: il s´agit d´une étude documentaire descriptive portant sur des dossiers d´enfants épileptiques suivis à la consultation externe de neurologie pédiatrique de l´HPZ pendant une période de 3 ans allant du 1^er^ janvier 2015 au 31 décembre 2018. La consultation et le suivi des enfants épileptiques sont assurés par le personnel médical du service de pédiatrie de l´HPZ. Le personnel est constitué d’un neuropédiatre assistant universitaire, de deux autres pédiatres praticiens hospitaliers. Ils reçoivent des enfants en phase critique et en phase inter critique venant des services de pédiatrie des deux hôpitaux de la commune de Ziguinchor, mais également d´autres structures de la région et de la sous-région (Gambie, Guinée Bissau). L´équipe de la pédiatrie de l´HPZ est aidée par le personnel du Centre Psychiatrique de Ziguinchor (CPZ) situé dans la même commune. Les enregistrements électro-encéphalographiques sont faits par deux techniciennes du CPZ et l´interprétation est par les médecins (le neuropédiatre et le psychiatre épileptologue).

**Population d´étude et critères de sélection**: l´étude concerne les enfants épileptiques suivis à la consultation externe de neurologie pédiatrique de l´HPZ. Critères d´inclusion: ont été inclus dans l´étude, tous les dossiers de patients âgés de moins de 15 ans, suivis à la consultation externe de neurologie pédiatrique de l´HPZ, dont le diagnostic d´épilepsie a été retenu. Critères de non inclusion: étaient exclus de l´étude, les dossiers incomplets ou les dossiers de patients n´ayant pas bénéficié d´électro-encéphalogramme.

**Collecte des données et paramètres d´intérêt**: pour tous les malades inclus dans l´étude, nous avons recueilli les données épidémiologiques et sociodémographiques; les données de la grossesse et de l´accouchement et les données électro-cliniques à partir d´une fiche d´enquête. Sur cette fiche, sont précisés : données épidémiologiques et sociodémographiques (âge et sexe de l´enfant) ; existence d´une consanguinité parentale ; origine géographique et niveau socioéconomique (NSE) des familles; a) anomalies de la grossesse et de l´accouchement, anomalies du développement psychomoteur ; existence d´une épilepsie dans la famille; b) données cliniques et électriques des crises et type de syndrome épileptique.

**Définitions opérationnelles**: dans l´origine géographique, le milieu urbain était représenté par la commune de Ziguinchor, le milieu sub urbain par les autres communes départementales et le milieu rural par les villages. Le NSE était évalué à partir des biens de la famille (télévision, frigo, voiture, téléphone, maison personnelle, accès à l´eau et l´électricité, profession des parents). Il peut être bas, moyen, bon. La consanguinité était définie comme suit: a) cousins et cousines germains sont liés au 1^er^degré, b) cousins et cousines issus de germains au 2^e^degré. La classification a été faite en fonction des critères de la classification internationale des épilepsies et syndromes épileptiques de 1989.

**Analyse statistique**: la saisie et l´analyse des données ont été réalisées avec les logiciels Microsoft Word 2019 et Epi Info 6.

**Considérations éthiques**: les règles d´anonymat et de confidentialité ont été respectées.

## Résultats

Durant la période de 3 ans nous avons admis 117 dossiers de patients suivis en consultation de neurologie pédiatrique, parmi lesquels 73 cas d´épilepsie, soit une fréquence de 62,4%. Le nombre de cas d´épilepsies colligées était de 55 (37 garçons et 18 filles); le reste des dossiers (18) était incomplets.

**Données épidémiologiques et socio démographiques**: l´âge moyen des enfants était de 4,3 ans [5 mois-13 ans]. Les enfants ayant un âge inférieur ou égal à 5 ans étaient les plus représentés (70,9%). Soixante pourcent (60%) des malades étaient originaire du milieu rural. Le niveau socioéconomique des familles était bas dans 67,3%. Huit (14,5%) enfants étaient issus d´un mariage consanguin. Le Tableau 1 résume la répartition des enfants selon les données épidémiologiques et socioéconomiques.

**Tableau 1:** répartition des patients selon les données épidémiologiques, sociodémographiques

Variables	Effectif (n=55)	Pourcentage (%)
**Age**		
5 mois – 2 ans 11 mois	20	36,4
3 – 5 ans 11 mois	19	34,5
6 – 10 ans 11 mois	13	23,6
11 – 13 ans	03	05,5
**Origine géographique**		
Urbaine	08	14,6
Sub urbaine	14	25,4
Rural	33	60,0
Bas	37	67,3
**Niveau socioéconomique**		
Moyen	15	27,3
Bon	03	05,4
**Consanguinité parentale**		
1^er^ degré	1	01,8
2^e^ degré	7	12,7

NSE*: niveau socio-économique

**Figure 1:**
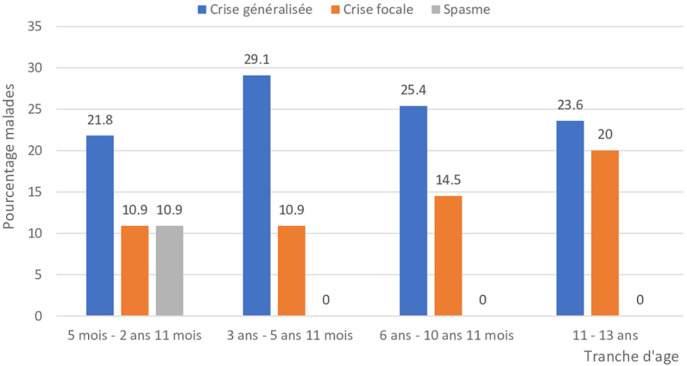
répartition des types de crises selon la tranche d´âge de l´enfant

**Données cliniques et paracliniques**: l´âge moyen des enfants au début de la maladie épileptique était de 2,5 ans [1 mois-12 ans]. Le délai moyen de consultation à la structure par rapport au début des crises était de 12 mois [2 jours-96 mois]. Trente-six virgule cinq pour cent (36,5%) des malades avaient commencé par un traitement traditionnel. Les crises d´épilepsie étaient généralisées dans 72,7% des cas et focales dans 27,3% des cas. Elles survenaient dans un contexte de poussée hyperthermique chez 08 enfants (soit 14,5%); d´émotion vive chez 06 enfants (10,9%); de manque de sommeil chez 07 enfants (12,7%) et de fatigue chez 13 enfants (23,6%). Les crises épileptiques prédominaient la nuit dans 15,6% des cas ; la veille dans 54,5% des cas et le reste (29,9% des cas) sans horaire fixe. La crise généralisée tonico-clonique représentait 43,6% des cas. Le Tableau 2 donne la répartition des enfants selon les types de crises. Les crises généralisées prédominaient quelle que soit la tranche d´âge. Leur pourcentage diminuait de façon inversement proportionnelle avec l´âge de l´enfant à partir du troisième anniversaire. La fréquence des crises focales augmentait de façon proportionnelle à l´âge de l´enfant. Elles représentaient 20% chez les enfants âgés entre 11 et 13 ans. Les spasmes étaient notés chez les enfants âgés de moins de 3 ans. La Figure 1 donne la répartition des types de crises selon la tranche d´âge. L´EEG était réalisé chez les 55 enfants (100%). Les résultats de l´EEG ont révélé des tracés de crises focales unilatérales chez 04 enfants (07,3%); focales bilatérales chez 09 enfants (16,4%); généralisées chez 26 enfants (47,3%) et normaux chez 16 enfants (29,1%). Le rythme de fond était normal chez 38 enfants (69,1%) et altéré chez 17 enfants (30,9%). Le scanner cérébral était réalisé chez 33 enfants. Il était normal chez 21 enfants (63,6%). Les principales anomalies retrouvées, étaient l´atrophie cortico-sous corticale dans 08 cas; la schizencéphalie dans 1 cas; l´hémimégalencéphalie dans 1 cas; l´hydrocéphalie dans 1 cas et l´ischémie cérébrale dans 1 cas.

**Tableau 2:** répartition des enfants selon le type de crise épileptique

Type de crise	Effectif (n=55)	Pourcentage (%)
**Crises généralisées (n=40 soit 72,7%)**	****	****
**Motrice**	**37**	**67,3**
Tonico clonique	24	43,6
Tonique	06	10,9
Myoclono astatique	01	01,8
Spasmes infantiles	06	10,9
**Non motrice**	**03**	**05,4**
Absences	03	05,4
**Crises Focales (CF) (n=15 soit 27,3%)**	****	****
**CF avec trouble conscience**	**10**	**18,2**
Tonico clonique	06	10,9
Tonique	02	03,6
Clonique	01	01,8
Myoclonique	01	01,8
**CF sans trouble conscience**	**04**	**07,3**
Tonico clonique	04	07,3
**CF secondairement généralisée**	**01**	**01,8**
Tonico clonique	01	01,8

**Données syndromiques**: sur les 55 patients concernés, 18 enfants présentaient une épilepsie idiopathique (32,7%), 17 enfants présentaient une épilepsie non idiopathique (30,9%) ; 08 enfants présentaient une convulsion fébrile (14,5%) et 12 enfants présentaient une épilepsie non classable. Le Tableau 3 résume la répartition des enfants selon les syndromes épileptiques.

**Tableau 3:** répartition des enfants selon les syndromes épileptiques

Syndrome épileptique	Effectif	Âge moyen de début (ans)	Consanguinité* n(%)	Epilepsie familiale n(%)
**Épilepsie idiopathique (n=18)**				
EGI*				
EAE*	03	05,5	00/03 (00,0)	02/03 (66,7)
EMJ*	01	13,0	01/01 (100,0)	00/01 (00,0)
EPI*				
EPR*	13	05,9	00/13 (00,0)	04/13 (30,8)
EPO*	01	04,4	00/01 (00,0)	00/01 (00,0)
**Épilepsie non idiopathique (n=17)**				
EGNI*				
West*	06	02,2	02/06 (33,3)	00/06 (00,0)
Doose*	01	03,4	00/01 (00,0)	00/01 (00,0)
AEE*	09	03,8	03/09 (33,3)	00/09 (00,0)
EPNI*				
SR*	01	11,0	00/01 (00,0)	01/01 (100,0)
**Convulsion fébrile (n=08)**	08	03,2	00/08 (00,0)	01/08 (12,5)
**Épilepsie inclassable (n=12)**	12	04,2	02/12 (16,7)	03/12 (25,0)

Consanguinité*=consanguinité de 1^er^ et de 2^e^ degré ; EGI : épilepsie généralisée idiopathique; EPI : épilepsie partielle idiopathique; EGNI : épilepsie généralisée non idiopathique; EPNI : épilepsie partielle non idiopathique EAE : épilepsie absence de l´enfant; EMJ : épilepsie myoclonique juvénile; EPR : épilepsie à paroxysme rolandique EPO : épilepsie à partielle occipitale; West : syndrome de West; Doose : syndrome de Doose; AEE : autres encéphalopathies épileptiques; SR : syndrome de Rasmussen

**Epilepsies idiopathiques (EI)**: dix-huit (18) enfants étaient suivis pour une EI. Une consanguinité parentale et des antécédents familiaux d´épilepsie étaient retrouvés respectivement chez 1 et 6 enfants (soit 05,5 et 33,3% des cas). Quatorze enfants présentaient une épilepsie partielle idiopathique représentée essentiellement par les épilepsies à paroxysmes rolandiques (13 enfants soit 92,8%). Quatre enfants présentaient une épilepsie généralisée idiopathique représentée essentiellement par les épilepsies absence de l´enfant, avec 03 cas.

**Epilepsies non idiopathiques (ENI)**: dix-sept (17) enfants étaient suivis pour une ENI. La consanguinité parentale et les antécédents familiaux d´épilepsie étaient retrouvés respectivement chez 5 et 1 enfant (soit 29,4 et 05,4% des cas). Les facteurs étiologiques étaient dominés par les anomalies de la grossesse et de l´accouchement (29,1%). Une asphyxie néonatale était retrouvée chez 18,1% des enfants (n=10), une prématurité et/ou un petit poids de naissance chez 05,4% des enfants (n=03). Le Tableau 4 résume les facteurs étiologiques des enfants présentant une ENI. Tous les 17 enfants du groupe des ENI, présentaient des troubles moteurs et/ou des troubles cognitifs. Les anomalies étaient dominées par les troubles posturo-moteurs (21,8%), du langage (09,1 %) et de la cognition (10,9 %). Les troubles cognitifs allaient d´un contact social pauvre chez les nourrissons à un déficit cognitif global chez les enfants plus âgés. Seize (16) enfants avaient une épilepsie généralisée non idiopathique (EGNI) dont 01 syndrome de Doose (06,2%) ; 06 syndromes de West (37,5%) et 09 autres encéphalopathies épileptiques (56,3%).

**Tableau 4:** répartition des enfants selon les facteurs étiologiques des épilepsies non idiopathiques

Facteurs étiologiques	Effectif (55)	Pourcentage (%)
**Anomalies de la grossesse et de l´accouchement**	**16**	**29,1**
Asphyxie néonatale	10	18,1
Prématurité et/ou petit poids de naissance	03	05,4
Ictère néonatal	02	03,6
Infection néonatale	01	01,8
**Encéphalopathie**	**09**	**16,4**
Suspicion de maladie métabolique	02	03,6
Post méningitique (méningite bactérienne)	01	01,8
Non étiquetée	06	10,9
**Autres facteurs étiologiques**	**11**	**20,0**
Convulsion dans un contexte de fièvre	04	07,3
Épilepsie dans la familiale	03	05,4
Accident vasculaire cérébral	01	01,8
Traumatisme crânien	01	01,8
**Autres**	02	03,6

## Discussion

L´épilepsie est une réalité en milieu pédiatrique. Dans notre étude, l´âge moyen des patients était de 4,3 ans et 70,9% de la population étaient des enfants d´âge inférieur ou égal à 5 ans. La fréquence de 62,4% de l´épilepsie dans la consultation de neurologie pédiatrique à l´HPZ, est en phase avec la survenue précoce de cette maladie. En effet selon une revue de la littérature, plus de 60% des cas d´épilepsies surviennent avant l´âge de 20 ans [5,6]. Dans l´étude de Ndiaye *et al*., l´âge moyen des patients était de 6,3 ans et 45% des enfants étaient âgés entre 1 et 6 ans [7]. Dans l´étude de Ngugi *et al*., la tranche d´âge de 0 à 5 ans représentait 50% de la population des patients épileptiques tout âge confondu [2]. Le sexe ratio garçons/fille de 2,0 retrouvé dans notre étude est superposable à ce qui est retrouvé dans la littérature [2,7,8]. Dans notre étude, 60% des enfants étaient originaire du milieu rural. Des résultats similaires étaient trouvés en 2001 par la ligue sénégalaise contre l´épilepsie, en collaboration avec le ministère de la santé dans son programme de plan quinquennal de lutte contre l´épilepsie.

La prise en charge de l´épilepsie chez l´enfant en Afrique subsaharienne souffre d´un gap thérapeutique très élevé, de 23 à 100% selon les régions [9,10]. Les données de la littérature révèlent qu´en Afrique subsaharienne, rares sont des pays qui possèdent un protocole national de prise en charge de l´épilepsie [11]. En outre, l´Afrique subsaharienne n´a toujours pas un accès facile, ni aux antiépileptiques les plus récents, ni aux autres possibilités thérapeutiques déjà largement utilisés dans les pays développés [12]. Ce gap de la prise en charge serait lié au manque de personnel qualifié, aux prix élevés des médicaments et/ou à leur indisponibilité ainsi qu´aux croyances culturelles mystico-religieuses qui entourent encore cette maladie. En effet, dans notre étude le délai moyen de consultation des malades à notre structure par rapport au début des crises était de 12 mois [2 jours-96 mois]. Trente-six virgule cinq pour cent (36,5%) des malades avaient commencé par un traitement traditionnel. Le type de crise le plus fréquemment rencontré dans notre étude est la crise généralisée (72,7% des cas) avec une prédominance de la forme tonico-clonique (43,6% des cas). Nos résultats sont comparables à ceux de plusieurs autres auteurs africains [13-15]. Ceci pourrait s´expliquer par la relative rareté des appareils EEG dans nos contextes et le caractère spectaculaire des crises généralisées. Une vidéo faite par les parents à domicile peut aider dans la classification des crises. Sur le plan syndromique, on note dans notre série d´épilepsies idiopathiques, une prédominance des épilepsies à paroxysmes rolandiques (EPR), des épilepsies-absence (EA). Cela est en conformité avec les données de la littérature: les EPR constituent le type le plus commun d´épilepsies partielles idiopathiques de l´enfant, avec 8 à 23% des épilepsies de l´enfant [7,16,17]. A Dakar, Ndiaye et al., retrouvaient les EPR dans 8,3% et les EAE dans 4,6% des épilepsies de l´enfant [7]. Dans une autre série faite toujours à Dakar, les auteurs notaient les EPR dans 22,0% des cas et les EAE dans 07,5% des cas d´épilepsies idiopathiques de l´enfant [8].

Dans les épilepsies non idiopathiques, l´épilepsie n´est souvent qu´un élément d´un ensemble plus complexe ou le handicap moteur et/ou cognitif est au premier plan. Dans les pays en voie de développement, du fait de la multiplicité des facteurs étiologiques et du sous-équipement sanitaire, une étiologie spécifique n´est mise en évidence que dans 40% des cas [5,18]. L´IRM, examen de choix dans l´exploration morphologique du cerveau chez les enfants épileptiques, n´est pas encore disponible à Ziguinchor, or 51,7% de nos patients porteurs d´une épilepsie non idiopathique avaient un scanner cérébral normal. L´étude des facteurs de risque, dans notre série, montre une nette prédominance des anomalies de la grossesse et de l´accouchement; 18,1% des enfants ont été réanimés à la naissance du fait d´une asphyxie. Ndiaye et al., avaient trouvé 14,4% dans une population de nourrissons à Dakar [7]. Dans une revue de la littérature sur l´épilepsie en Afrique sub-saharienne, Ngoungou *et al*., estiment que les causes périnatales peuvent être considérées comme majeures, même si les estimations sont très variables, 1-36% des cas [6]. Dans notre pays, du fait de l´insuffisance des ressources humaines, 21% des femmes accouchent à domicile sans l´assistance d´un personnel de santé et parmi celles qui accouchent dans les structures de santé, nombreuses sont celles qui sont assistées par les « matrones », personnel communautaire de base sans qualification, recruté et formé sur le tas pour faire face au déficit structurel en ressources humaines [19].

## Conclusion

L´épilepsie représente 62,4% de la consultation de neurologie pédiatrique de l´Hôpital de la Paix de Ziguinchor. C´est une affection qui prédomine en milieu rural chez les garçons de moins de 5 ans, issus des familles défavorisées. Les crises généralisées tonico-cloniques et les crises focales avec trouble de la conscience sont les tableaux cliniques les plus fréquentes. Les syndromes épileptiques sont dominés par l´épilepsie à paroxysme rolandique ; l´épilepsie absence et le syndrome de West. Les facteurs étiologiques des épilepsies non idiopathiques sont dominés par les anomalies de la grossesse et de l´accouchement.

### Etat des connaissances sur le sujet

- L´épilepsie pose un problème de santé publique en Afrique subsaharienne; Elle concerne principalement les enfants;
- Au Sénégal, l´incidence et la prévalence de l´épilepsie sont mal évaluées faute de données générales et surtout régionales;
- Dans la région de Ziguinchor, sud du Sénégal, la stigmatisation de la maladie, le manque de neuropédiatres, la quasi absence des appareils d´électroencéphalogramme expliquent ce manque de données.

### Contribution de notre étude à la connaissance

- Données hospitalières sur l´épilepsie de l´enfant à Ziguinchor;
- Profils épidémiologiques ; électro-clinique et syndromiques de l´épilepsie de l´enfant à Ziguinchor;
- Améliore la prise en charge de l´épilepsie de l´enfant à Ziguinchor.
